# Cerebrospinal fluid proteomic profiling reveals potential biomarkers and altered pathways in myotonic dystrophy type 1

**DOI:** 10.3389/fnins.2025.1709678

**Published:** 2025-11-26

**Authors:** Marwa Zafarullah, Tahereh Kamali, Katharine A. Hagerman, Lisa Ghiglieri, Tina Duong, Eric Wang, Jacinda B. Sampson, John W. Day

**Affiliations:** 1 Department of Neurology and Neurological Sciences, Stanford School of Medicine, Stanford, CA, United States; 2 Center for Neurogenetics and Department of Molecular Genetics and Microbiology, College of Medicine, University of Florida, Gainesville, FL, United States; 3 Department of Neurology, Stanford Health Care, Stanford, CA, United States

**Keywords:** myotonic dystrophy type 1, proteomics, potential cerebrospinal fluid signatures, protein pathways, neuromuscular disorders

## Abstract

**Introduction:**

Myotonic dystrophy (DM), the most common adult-onset muscular dystrophy, affects not only motor function and muscle integrity but also leads to debilitating cardiopulmonary, gastrointestinal, and multisystem complications. Central nervous system (CNS) involvement is increasingly recognized, manifesting as impairments in working memory, executive function, sleep regulation, and mood and behavior. These interrelated, multisystemic features contribute to multifaceted symptoms that significantly reduce quality of life for patients and their families. To identify potential biomarkers of CNS disease activity in DM1, we performed the first exploratory cerebrospinal fluid (CSF) proteomic profiling study.

**Methods:**

CSF samples from patients with DM1 (*n* = 11) and healthy controls (*n* = 5) were analyzed using Olink monoclonal antibody panels, quantifying 1,072 proteins. LASSO (Least Absolute Shrinkage and Selection Operator) regression identified proteins discriminating between DM1 and controls. Pathway enrichment analysis was performed using the Reactome database to assess biological significance.

**Results:**

Six candidate biomarker proteins were differentially expressed between between DM1 patients and controls: CKAP4, SCARF1, NCAM1, CD59, PTH1R, and CA4. LASSO analysis further identified 15 proteins discriminating DM1 and controls, implicating pathways related to neuronal health, neuroinflammation, cognitive impairment, skeletal abnormalities, motor control, neuromuscular junction integrity, and cytoskeletal regulation. Dysregulated pathways included IGF transport, MAPK signaling, NCAM signaling, and broader signal transduction cascades pathways also implicated in other neurodevelopmental, neurodegenerative, and neuromuscular disorders.

**Discussion:**

This first exploratory CSF proteomic analysis in DM1 identified dysregulated protein networks that may underlie CNS dysfunction in this multisystemic disease. These findings provide novel insights into DM1 pathophysiology and support the potential of CSF proteomic signatures as candidate diagnostic tools, indicators of disease activity, and measures of therapeutic response, pending validation in larger, independent cohorts.

## 1 Introduction

Myotonic dystrophy type 1 (DM1) represents the most prevalent form of muscular dystrophy in adults, affecting approximately one in every 2,100 births worldwide (Johnson et al., 2021). This complex multisystem disorder presents with a heterogeneous array of clinical manifestations, encompassing myotonia, progressive muscle wasting, insulin resistance, posterior cataracts, gastrointestinal and cardiac abnormalities, as well as neuropsychiatric disturbances (Day and Ranum, 2005). The central nervous system (CNS) manifestations are particularly debilitating, featuring intellectual disability, executive dysfunction, attention deficits, and centrally mediated fatigue and sleep disorders, which collectively contribute to significant functional impairment in affected individuals. At the molecular level, DM1 caused by an expansion of CTG trinucleotide repeats within the 3′ untranslated region of the dystrophia myotonica protein kinase (DMPK) gene on chromosome 19, where disease severity demonstrates a direct correlation with the number of repeats (Hunter et al., 1992; Harley et al., 1993; Redman et al., 1993; Morales et al., 2012). These expanded repeats lead to an RNA gain-of-function, whereby toxic RNA forms hairpin loop structures that sequester essential RNA-binding proteins (RBPs), particularly MBNL factors, thereby disrupting normal RNA splicing and downstream cellular signaling cascades (Mankodi et al., 2000; Ho et al., 2004). Additionally, hyperphosphorylation of CUGBP1 impairs its function and contributes to widespread dysregulation of key signaling pathways and post-translational modifications (Ward et al., 2010). Despite decades of research elucidating these pathogenic mechanisms, no therapeutic interventions currently exist to halt or slow DM1 progression, underscoring the urgent need for reliable biomarkers and the identification of altered signaling pathways. These biomarkers are crucial for evaluating the effectiveness of new treatments and the understanding of dysregulated signaling mechanisms will facilitate the development of more effective and targeted therapeutic strategies.

Proteomics has emerged as a powerful tool for identifying potential biomarkers, uncovering disrupted signaling pathways, and providing insights into protein post-translational modifications in diseases like DM1. Recent investigations leveraging multi-omics strategies, including comprehensive proteomic analyses, have provided significant insights into the pathological mechanisms underlying DM1 in both murine models and human patient samples. Notably, Hernández and colleagues identified upregulation of RAB3A and hyperphosphorylation of synapsin I in DMSXL mice, establishing a direct link between synaptic protein dysregulation and the neurological manifestations characteristic of DM1 (Hernández-Hernández et al., 2013). Complementing these findings, proteomic analysis revealed significant downregulation of the glutamate transporter GLT1, resulting in enhanced neurotoxicity in both DMSXL mice and human patients due to MBNL depletion (Sicot et al., 2017). Furthermore, the integration of phospho-proteomics with advanced cell imaging techniques has illuminated the profound impact of expanded CUG RNA on glial cell metabolism in DMSXL astrocytes (González-Barriga et al., 2021), while Solovyeva et al. employed combined transcriptomic and proteomic approaches to identify novel disease-associated genes in the HSALR mouse model (Solovyeva et al., 2024).

Recent clinical investigations have successfully utilized unbiased proteomic profiling to identify dysregulated proteins in DM1 fibroblasts, including CAPN1 and CTNNB1, which are modulated by GSK3β, thereby demonstrating the clinical utility of proteomics for disease monitoring (Grande et al., 2021). Additionally, comprehensive proteomic profiling across human fibroblasts, murine skeletal muscle, and cardiac tissue has revealed that biomarkers such as periostin are intimately linked to cardiac dysfunction and overall disease severity in DM1 (Nguyen et al., 2023). Muscle biopsy studies have further identified proteins associated with myogenesis and muscle function, suggesting promising therapeutic targets for ameliorating muscle pathology in DM1 patients (Aoussim et al., 2023). In the landmark OPTIMISTIC trial, proteomic analysis of blood samples revealed specific proteins that correlated with CTG repeat length and physical activity levels, offering potential biomarkers for tracking disease progression and therapeutic response ('t Hoen et al., 2023).

The utility of cerebrospinal fluid (CSF) proteomics for biomarker discovery has been extensively demonstrated across various neurological disorders, including spinal muscular atrophy (Beaudin et al., 2023), Alzheimer’s disease (Gaetani et al., 2021; Dammer et al., 2022), bipolar disorder (Göteson et al., 2021; Isgren et al., 2022), and multiple sclerosis (Huang et al., 2020; Rabin et al., 2024). However, the present study represents a first-ever investigation into the application of CSF proteomics for identifying potential biomarkers and elucidating disrupted protein signaling pathways specifically in DM1. In this investigation, we performed Olink proteomic analysis of CSF samples from 11 DM1 patients and 5 healthy controls to systematically examine the central nervous system pathophysiology of DM1 and identify potential biomarkers. A secondary objective was to characterize dysregulated protein pathways, which may reveal shared pathophysiological mechanisms with other neuromuscular disorders and provide insights into common therapeutic targets.

## 2 Materials and methods

### 2.1 Study participants

We conducted comprehensive proteomic profiling of 11 participants diagnosed with DM1, aged 25–54 years, and 5 non-carrier healthy controls (HCs). All studies and protocols were performed in compliance with the Institutional Review Board at Stanford University. Written informed consent was obtained from all participants before study participation, in accordance with the Declaration of Helsinki (Version 2013) and other applicable regulatory requirements. All DM1 diagnoses were genetically confirmed by molecular testing demonstrating CTG repeat expansions in the DMPK gene on chromosome 19q13.3.

### 2.2 CSF sample preparation and proteomic profiling

CSF was collected from the patient in accordance with the approved protocol. The initial 0.5 mL of CSF was discarded to minimize potential contamination from blood or tissue introduced during needle insertion. Subsequent CSF fractions were collected; a small portion was used for routine cell count and biochemical testing to confirm sample quality, while the remaining CSF underwent centrifugation at 1,000 rpm for 15 min at 4 °C to pellet the cellular components. Subsequently, aliquots of the supernatant were transferred to cryovials and frozen at −80 °C for proteomic analyses. Proteomic analysis was performed using the Olink ® platform (Uppsala, Sweden), with results reported in the Normalized Protein expression (NPX) scale, an arbitrary unit on a Log2 scale. The study utilized the Olink® 15 panels including cardiometabolic, cardiovascular II, cardiovascular III, cell regulation, development, immune response, inflammation, metabolism, neurology, oncology II, organ damage, and neuro exploratory panels, which collectively identified 1,113 proteins. The amplified sequences were detected and quantified using standard real-time PCR. Protein levels were normalized using Olink’s Intensity Normalized (v2) procedure, while data from the Neuro Exploratory panel^1^ employed the Inter-Plate Controls (IPC) normalization method due to its bimodal distribution. For quality control, four internal Olink® controls were included in each sample to ensure assay performance and sample integrity. Quality assessments were conducted in two phases: first, we checked that the standard deviation (SD) of the internal controls was below 0.2 NPX for each sample. Second, we ensured that the deviation of the internal control concentration from the median value was less than 0.3 NPX.

### 2.3 Data analysis

CSF proteomic data from both DM1 patients and control subjects were preprocessed to ensure data quality. We filtered the data to remove low-quality entries, defined as proteins with detection in fewer than 80% of samples, signal intensities below the platform’s limit of detection, or high technical variability (CV > 20%), retaining only proteins with consistent and reliable measurements. Differential expression analysis (Smyth, 2004) was conducted using linear models (limma package) with the design ~ group + sex, treating group (DM1 vs. HC) as the main effect and sex as a covariate. For each protein, we calculated mean expression levels across groups and derived log2 fold changes (log2FC) for interpretability. Two-sample *t*-tests were performed, and multiple testing correction was applied using the Benjamini-Hochberg method (Benjamini and Hochberg, 1995). Proteins with False Discovery Rate (FDR)-adjusted *p*-values < 0.05 were considered significantly differentially expressed. In addition, to ensure data reliability, proteins with more than 25% missing NPX values across samples were excluded. This filtering step was applied after initial exploratory analysis to examine their correlations and potential interactions. Box plots were created for each differentially expressed protein to visually compare DM1 and control groups, highlighting medians, interquartile ranges, and outliers, with significant proteins annotated for *p*-values below 0.05 (group effects are sex-adjusted). LASSO regression (Bakochi et al., 2021), a regularized regression method, was applied to identify proteins predictive of group classification (DM1 vs. controls), including sex as an additional predictor. As a sensitivity analysis, proteins were revisualized for sex and LASSO was re-run on the residuals, yielding consistent results. Proteins with absolute feature importance greater than 0.3 (|*β*| > 0.3) were considered significant contributors. In addition, only proteins with missing values in fewer than 25% of samples were retained to ensure data reliability. Fifteen proteins were selected by LASSO regression based on their predictive contribution to the model’s ability to classify samples correctly. Statistical significance of LASSO-selected proteins was subsequently assessed using two-sample *t*-tests with Benjamini-Hochberg correction for multiple testing. Among these, four proteins overlapped with the six statistically significant proteins identified through differential expression analysis, highlighting their dual role as both statistically robust and predictive candidate biomarkers. To interpret the biological relevance of differentially expressed proteins, pathway enrichment analysis was performed using curated gene sets from Reactome. Pathways with FDR-adjusted *p*-values < 0.05 were considered significant, and results were visualized in bar plots representing the top enriched pathways associated with DM1. Pathway enrichment results were validated by recalculating *p*-values and applying cross-validation techniques to ensure statistical robustness. A volcano plot was generated to present the distribution of differentially expressed proteins, with log2FC values plotted against −log10 *p*-values to highlight proteins with both high statistical significance and large expression changes. Proteins surpassing significance thresholds were visually emphasized as potential biomarker candidates.

### 2.4 Statistical power analysis

To assess the statistical power of our study given the small sample size, we conducted post-hoc power analyses for all differentially expressed proteins using observed effect sizes, sample sizes (*n* = 11 DM1, *n* = 5 HC), and *α* = 0.05. Cohen’s d effect sizes were calculated from mean differences and pooled standard deviations for each protein. Power calculations were performed using the pwr.t.test function in R (pwr package version 1.3–0). For the six significantly differentially expressed proteins, power ranged from 38% (CA4, Cohen’s *d* = 1.0) to 82% (CD59, Cohen’s *d* = 2.1), indicating adequate power (≥80%) only for proteins with very large effect sizes. Proteins with moderate effect sizes showed power ranging from 38 to 65%, reflecting the exploratory nature of this investigation and underscoring the need for validation in larger cohorts. The complete power analysis results are provided in Supplementary Table S1.

## 3 Results

**Figure 1:**
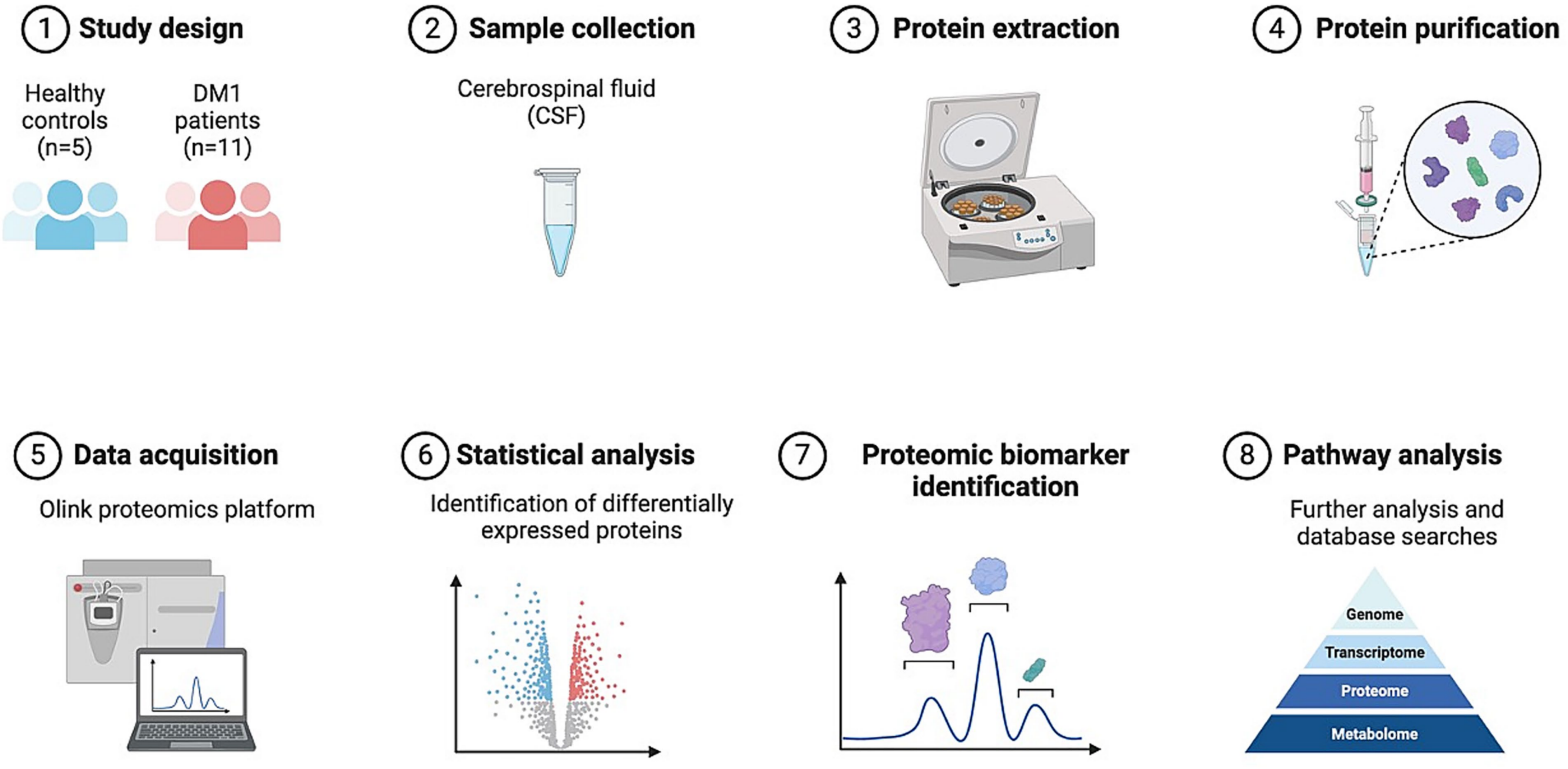
Study strategy and schematic illustration of CSF Olink proteomics. CSF collected from patients with DM1 (*n* = 11), and HC (*n* = 5) was used for Olink multiple panels. Subsequent bioinformatics analysis was performed to identify differentially expressed proteins, and pathway analysis.

We conducted a comprehensive analysis comparing proteomic profiles in the CSF of DM1 participants versus healthy controls to identify differentially expressed proteins and novel pathways using the Olink platform (Figure 1).

### 3.1 Demographics

**Figure 2:**
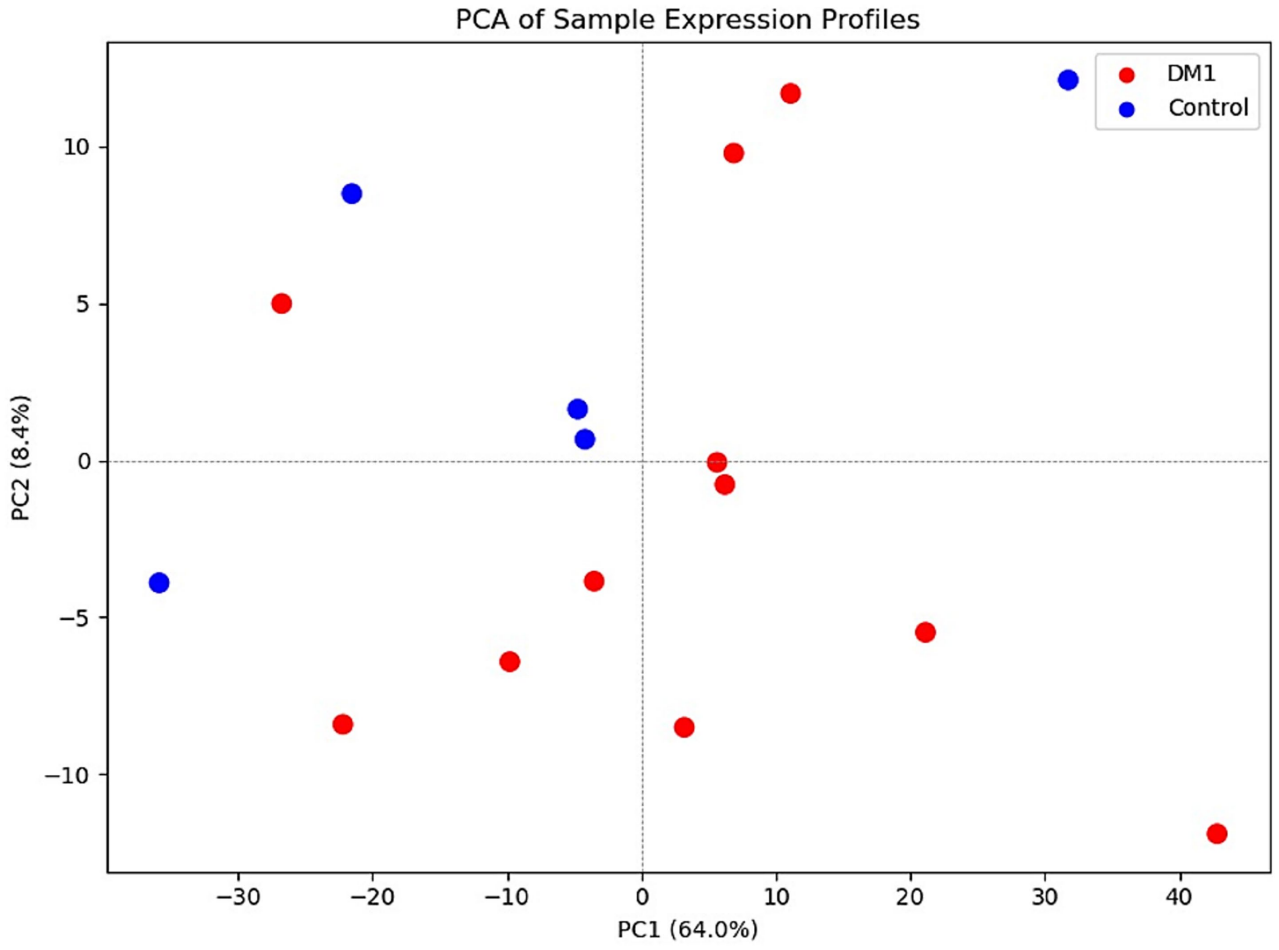
Principal Component Analysis (PCA) of DM1 and control samples shows partial group separation: PC1 accounts for 64% of the total variance. There is partial separation between the two groups along PC1, with most DM1 samples clustering on the right and most control samples clustering on the left or in the lower corner. A few samples overlap in the center, indicating mild heterogeneity or shared signaling between the groups.

A total of 16 participants were included in the study: 11 individuals with myotonic dystrophy type 1 (DM1) and 5 age- and sex-matched healthy controls (CTL) (Table 1). The DM1 group ranged in age from 25 to 54 years (mean age: 36.1 ± 9.8 years), with a predominance of females (10 females, 1 male). Reported CTG repeat sizes ranged from 75 to over 1,000, including one individual with a general DMPK expansion without a specific repeat count. Clinical phenotyping revealed varying disease severity among DM1 participants: most individuals (9/11) presented with distal muscle involvement, while one had combined distal and proximal weakness. No participants showed isolated proximal involvement. Regarding functional status, five participants were independently mobile, while six required assistances. Hypersomnia was reported in 5 of the 11 DM1 participants. The control group consisted of four males and one female, aged between 30 and 62 years (mean age: 44.4 ± 12.3 years). All control participants were neurologically healthy with no known neuromuscular disorders. Principal component analysis showed that PC1 explains 64% of the total variance, with partial separation between DM1 and control samples along PC1. Most DM1 samples clustered to the right, while most control samples clustered to the left. A few overlapping samples in the center suggest mild heterogeneity or shared signal (Figure 2).

**Table 1:** Demographics of the study participants.

Clinical severity					5	4	3	2	1	5 cDM - Severe	4 cDM - Mod	3 Moderate adult		2 Mild adult		1 Normal
	Condition	Age	Sex	CTG Repeats	Prox - Mod Severe	Distal + Proximal	Only distal	Min - Myotonia	Normal	Not independent	Independent	With hypersomnia	No hypersomnia	With hypersomnia	No hypersomnia	
Subject 1	DM1	25	F	500			X					X				
Subject 2	DM1	54	F	75				X							X	
Subject 3	DM1	44	F	542			X					X				
Subject 4	DM1	42	F	129			X									X
Subject 5	DM1	32	F	368			x							x		
Subject 6	DM1	32	F	250–350				X								X
Subject 7	DM1	26	F	536			X							X		
Subject 8	DM1	31	M	DMPK expansion		X										X
Subject 9	DM1	26	F	DMPK expansion			X							X		
Subject 10	DM1	54	F	714	X									X		
Subject 11	DM1	41	F	1,005			X							X		
Subject 12	CTL	62	M													
Subject 13	CTL	33	F													
Subject 14	CTL	55	M													
Subject 15	CTL	42	M													
Subject 16	CTL	30	M													

DM1, Myotonic Dystrophy Type 1; Y, Years; CTG, cytosine, thymine, and guanine.

CTG repeat sizes were obtained from clinical genetic testing reports when available. Precise repeat counts were not uniformly available for all participants, precluding correlation analyses between repeat length and proteomic findings. One participant had documentation of DMPK expansion without specific repeat quantification.

### 3.2 CSF proteomic profile identified differentially expressed proteins in DM1

**Figure 3:**
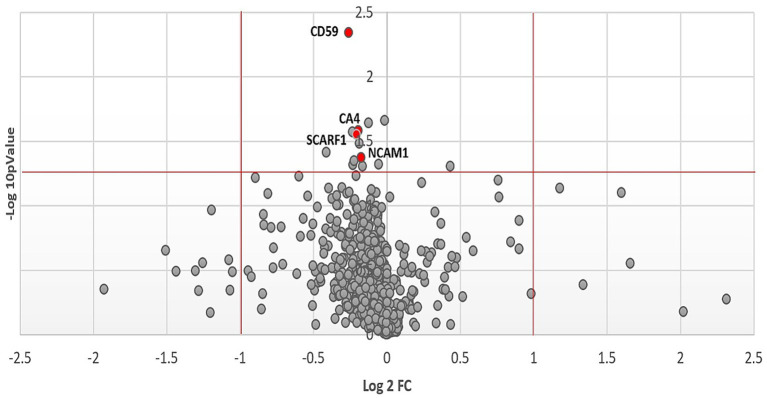
Volcano plot of proteomic analysis showing CSF proteomic changes between DM1 and HC. Log2 of the fold change is represented on the x-axis and −log10 of the *p* value on the y-axis. The horizontal red line indicates *p* = 0.05 and the vertical red lines indicate a fold change greater than 1 or inferior to −1.

**Figure 4:**
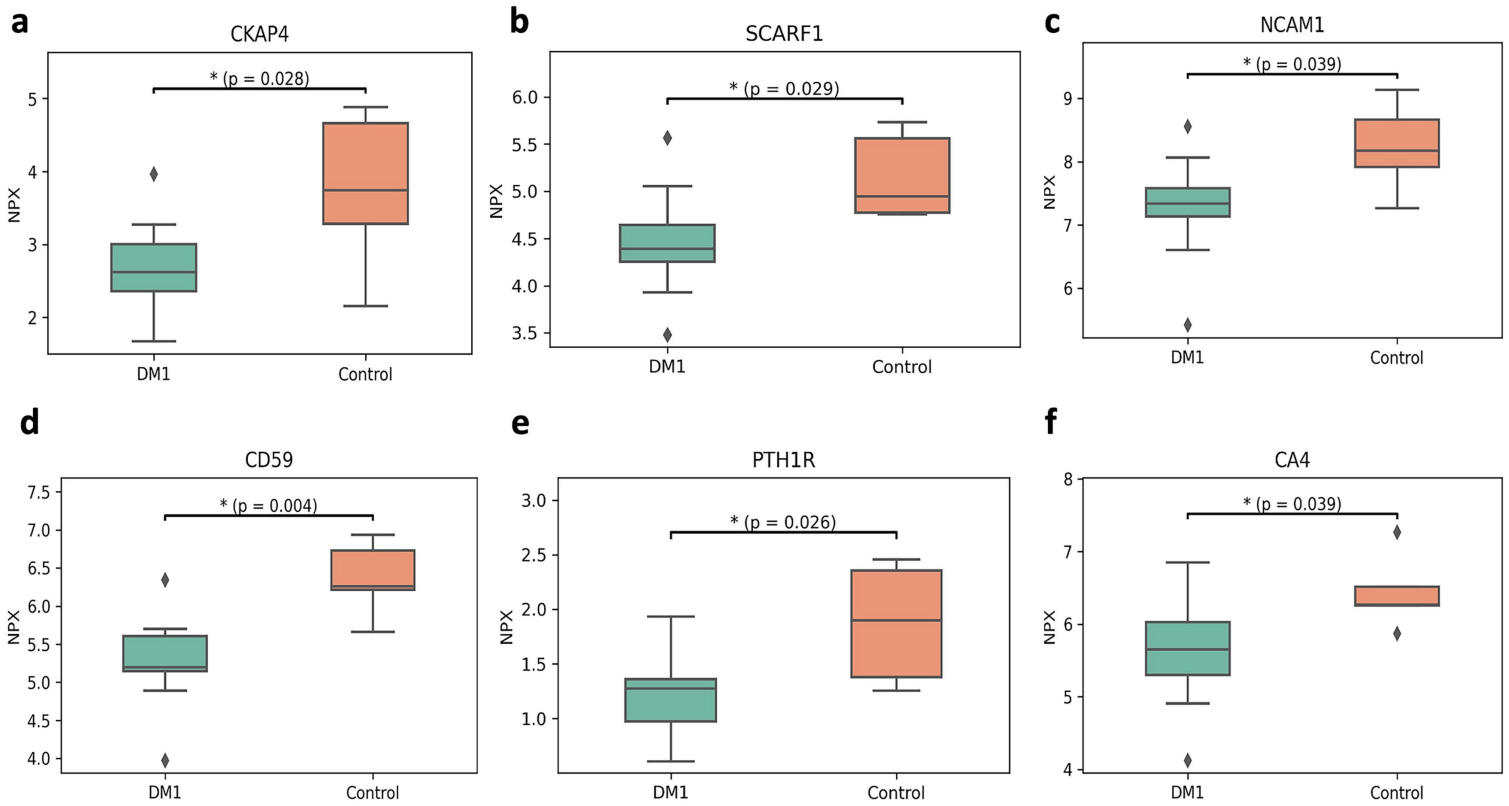
Significantly expressed proteins between DM1 and TD groups. Box plots showing decreased levels of CKAP4 **(a)**, SCARF1 **(b)**, NCAM1 **(c)**, CD59 **(d)**, PTH1R **(e)**, and CA4 **(f)** in DM1 as compared to TD. The heavy line in each box represents the median, the lower and upper box edges represent the 25th and 75th percentiles, respectively, and the lower and upper whiskers represent the smallest and largest observations, respectively. Statistically significant differences (*p* < 0.05) are marked with a*.

**Figure 5:**
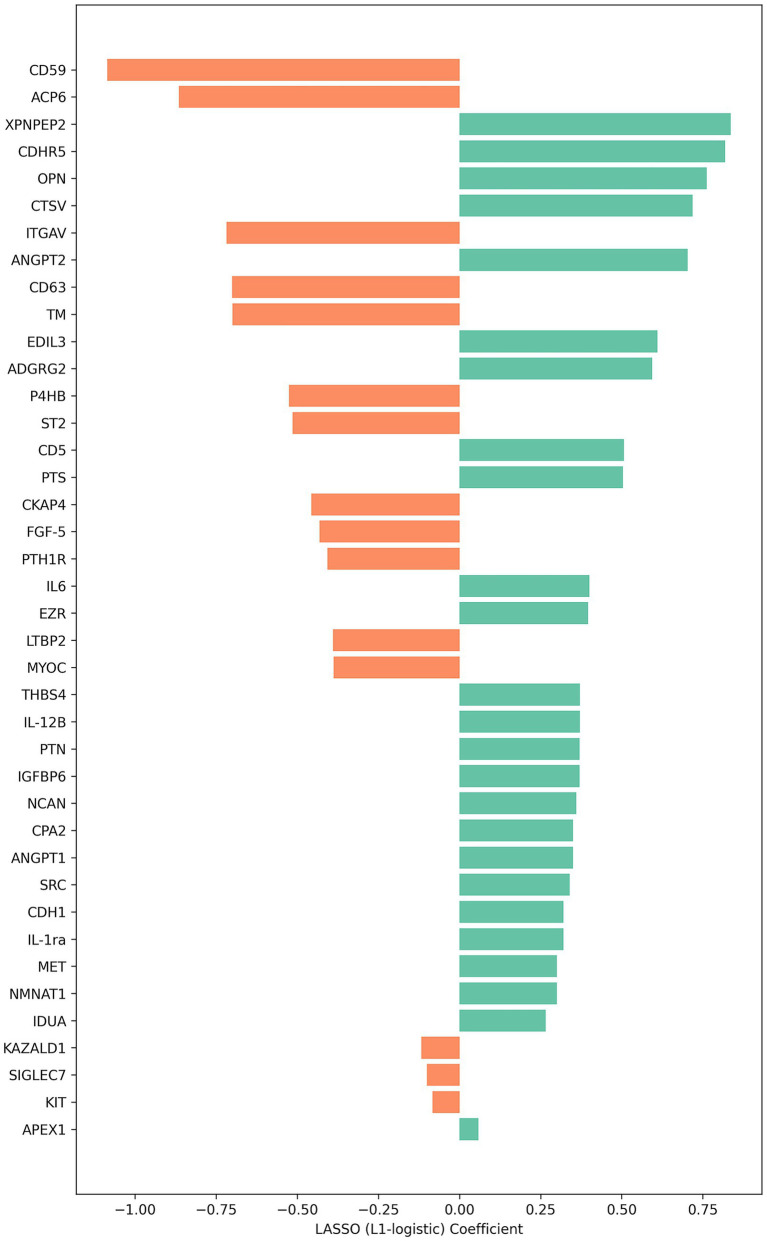
Proteins identified by LASSO logistic regression distinguishing DM1 from control samples. Bar plot displaying the LASSO (L1-regularized logistic regression) coefficients for proteins retained in the final model. The magnitude and direction of each coefficient indicate the relative contribution of each protein to group classification, with larger absolute values representing stronger predictive influence.

**Figure 6:**
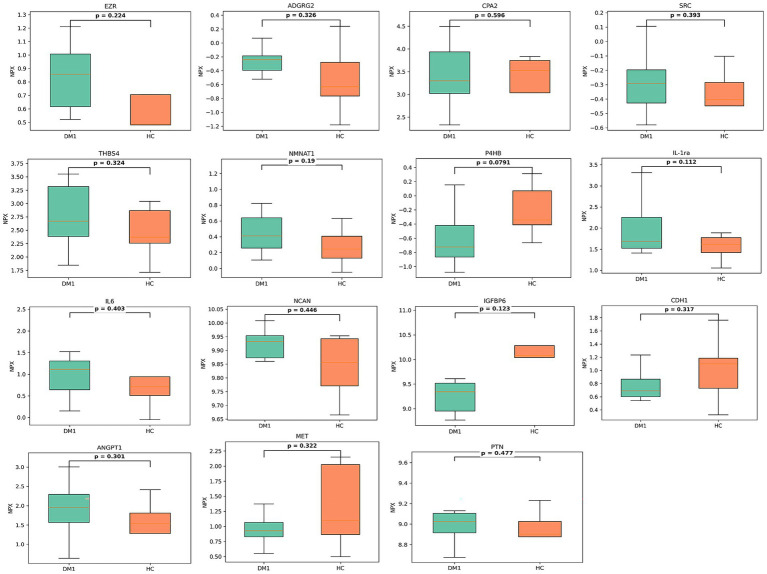
Box plots of proteins selected using LASSO regression for differentiation between DM1 patients and controls. The box plots display the distribution of expression levels for each selected protein in DM1 patients compared to controls. The heavy line in each box represents the median, the lower and upper box edges represent the 25th and 75th percentiles, respectively, and the lower and upper whiskers represent the smallest and largest observations, respectively. Statistically significant differences determined by two-sample *t*-tests with Benjamini-Hochberg correction for multiple testing are marked with asterisks (*p < 0.05, **p < 0.01, ***p < 0.001). These proteins showed significant differences between the two groups, indicating their potential as candidate biomarkers for distinguishing DM1 from healthy controls.

The Olink multiple panel global proteomic profile identified a total of 1,072 proteins in the comparison between DM1 and HC groups (Figure 3). Six candidate biomarker proteins CKAP4, SCARF1, NCAM1, CD59, PTH1R, and CA4 exhibited significant differential expression (*p* < 0.05) between the two groups. All six proteins were downregulated in DM1 CSF (Figure 4). LASSO regression analysis identified 15 candidate proteins that distinguished the two groups (Figure 5). Eleven proteins EZR, ADGRG2, CPA2, SRC, THBS4, NMNAT1, P4HB, IL-1ra, IL-6, NCAN, ANGPT1, and PTN were upregulated in DM1, while three proteins IGFBP6, CDH1, and MET were downregulated in DM1 compared to HCs (Figure 6).

### 3.3 Enrichment analysis identifies altered pathways between DM1 and HCs

**Figure 7:**
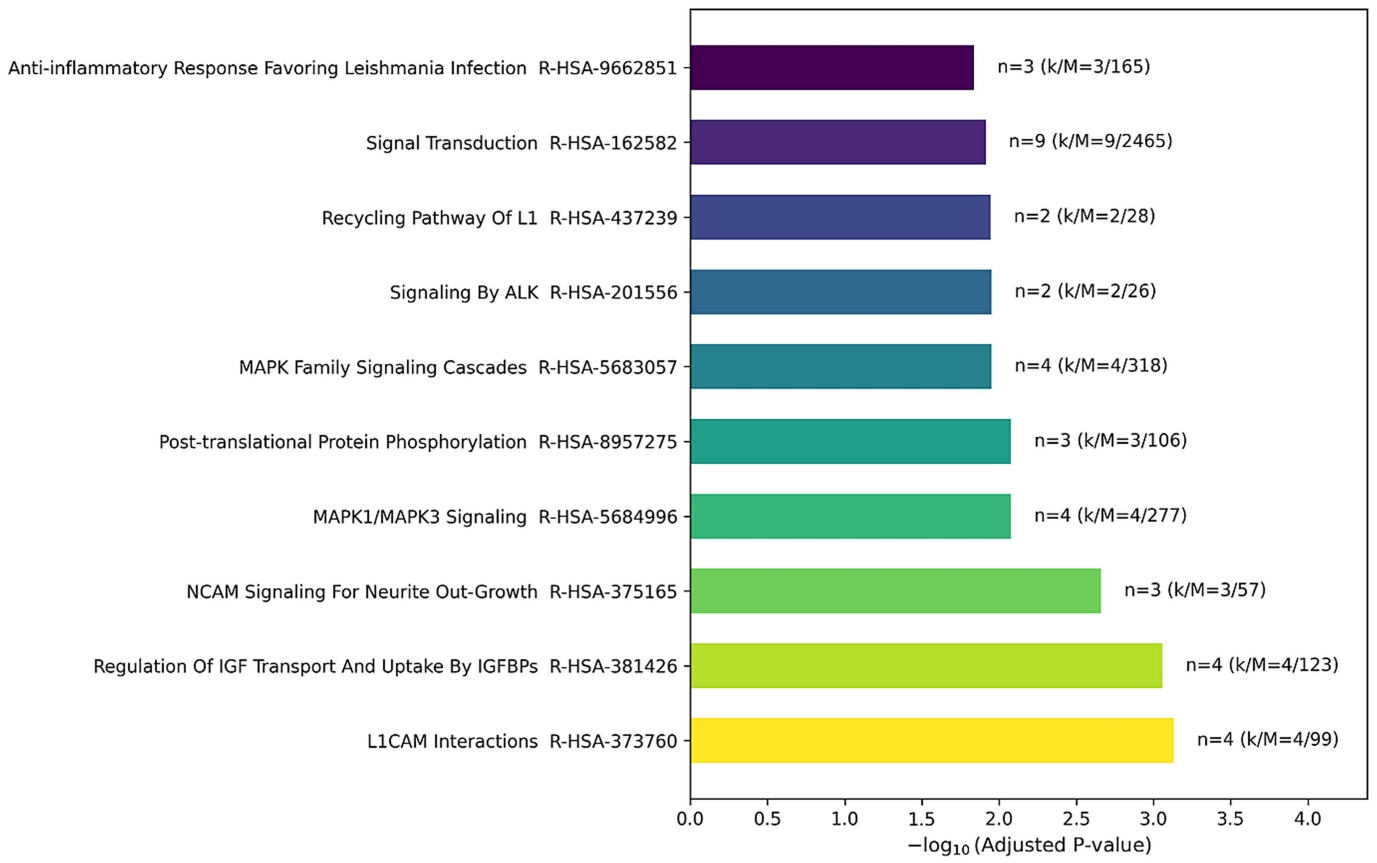
Top 10 reactome pathway enrichment analysis for DM1 using CSF proteomic data. The bar chart shows the −log10 (adjusted *p*-value) for each pathway, with pathways grouped and colored based on their −log10 (adjusted *p*-value). The analysis highlights key pathways involved in the condition, with the highest significance indicated by longer bars.

Reactome enrichment analysis (Figure 7) revealed significant differences in protein expression levels across various pathways between DM1 and HC groups. Notable pathways included IGF transport, MAPK signaling, L1CAM, NCAM, and post-translational protein phosphorylation signaling, underscoring their potential role as biological processes implicated in DM1 pathophysiology.

## 4 Discussion

In this exploratory analysis of DM1 cerebrospinal fluid (CSF) proteomic profiles, we identified six proteins that were significantly downregulated in DM1 compared to healthy controls (HCs) using differential expression analysis. Additionally, LASSO regression identified 15 proteins that distinguished DM1 from controls. These proteins represent diverse pathways involved in neuronal health, neuroinflammation, neuroprotection, neuronal survival, neuronal growth, synaptic plasticity, cognitive impairment, and cytoskeletal regulation. Enrichment analysis revealed several significantly dysregulated pathways associated with DM1, including IGF transport, MAPK signaling, L1CAM, and NCAM. These findings underscore the value of integrating proteomic profiling with advanced predictive modeling to identify biological processes involved in DM1 pathophysiology.

Our CSF proteomic findings complement recent multi-omics investigations in DM1, offering CNS-specific insights and revealing convergent disease mechanisms across molecular levels. Braun et al. (2022) demonstrated that RNA interference and CUG repeat-derived siRNAs drive transcriptomic dysregulation in DM1, influencing individual phenotypes. While their work highlights RNA-level mechanisms, our proteomic analysis captures downstream protein-level effects within the CNS, reinforcing the dysregulation of pathways such as MAPK signaling and cytoskeletal organization as central to DM1 pathology. Similarly, a large-scale serum proteomics study in 437 DM1 samples from the OPTIMISTIC trial, identifying 161 proteins associated with CTG repeat length (Van As et al., 2025). Their results revealed systemic immune abnormalities, including hypogammaglobulinemia, which align with our observation of CNS-specific immune alterations (elevated IL-6, IL-1ra, and reduced CD59). While serum proteomics correlated with motor function measures, our CSF biomarkers may reflect CNS-related symptoms such as cognitive and sleep disturbances. The limited protein overlap between biofluids underscores the importance of compartment-specific analyses and supports multi-compartment biomarker strategies. Finally, alignment with the proteogenomic study in DM1 mouse models further validates our findings, demonstrating shared dysregulated proteins and pathways across species. Together, these cross-platform and cross-compartment observations strengthen the evidence for convergent molecular mechanisms in DM1 and highlight potential therapeutic targets within both systemic and CNS domains (Solovyeva et al., 2024).

We demonstrate significant decreases in CKAP4 expression in DM1 CSF, consistent with previous research on cytoskeletal proteins in neurodegenerative disorders. This reduction suggests potential disruption in microtubule stability, impaired axonal transport, and compromised neuromuscular junction function. Additionally, decreased CKAP4 may contribute to neuroinflammation, increased neuronal vulnerability, and impaired synaptic plasticity, all of which could exacerbate the neurological and cognitive impairments observed in DM1 patients. These findings align with prior research highlighting the role of cytoskeletal proteins in neuronal function and their dysregulation in neurodegenerative disorders such as Friedreich’s ataxia, spinocerebellar ataxia type 5, Alzheimer’s disease, and Parkinson’s disease (Zhang et al., 2015; Bamburg and Bernstein, 2016; Piermarini et al., 2016; Avery et al., 2017; Del Rey et al., 2018; Muñoz-Lasso et al., 2020). Furthermore, CKAP4’s role in regulating transcription factors like FOXM1, which are involved in DNA repair through AKT/ERK signaling pathways associated with somatic polynucleotide repeat expansion CKAP4-mediated activation of FOXM1 via phosphorylation pathways regulates malignant behavior of glioblastoma cells (Xu et al., 2023), supports that CKAP4 dysregulation may underlie the cellular dysfunction observed in DM1. We also observed downregulation of SCARF1 in DM1 patients, which may indicate impaired neuroinflammatory responses, as SCARF1 is critical for clearing apoptotic cells and reducing inflammation (Ramirez-Ortiz et al., 2013). Similarly, we identified significantly reduced NCAM1 expression in DM1, consistent with previous studies linking decreased NCAM1 levels in plasma to neurological impairments, including autism spectrum disorder (ASD) (Yang et al., 2019), and its role as a sensitive biomarker in sera for Charcot–Marie–Tooth disease (CMT) (Jennings et al., 2022). In DM1, lower NCAM1 expression may contribute to impaired neuronal adhesion, disrupted synaptic plasticity, and reduced neuroplasticity, all of which can lead to cognitive and memory deficits. However, we did not directly assess their functional role within this cohort and from an exploratory perspective, these proteins may serve as candidates for further investigation in larger cohorts, particularly to link expression changes to functional outcomes such as cognitive measures for NCAM1 or developmental pathways for MAPK the future studies integrating functional assays will be needed to clarify these relationships.

A key strength of our study is the use of LASSO regression, which effectively handles high-dimensional CSF proteomic data by selecting the most relevant proteins that differentiate DM1 patients from HCs while mitigating overfitting risk. Among the 15 proteins upregulated in DM1 including EZR, ADGRG2, CPA2, SRC, THBS4, and IL-6 we observed that Thrombospondin-4 (THBS4) is significantly upregulated in DM1 CSF. Düzenli et al. (2022) provide evidence for TSP4’s role in neuropathic pain development, suggesting that its blockade could be a promising therapeutic approach. Additionally, THBS4 is involved in cell adhesion and cytoskeletal organization (Lawler et al., 1993) and has been reported as a potential biomarker for spinal muscular atrophy (SMA), with reduced levels found in pediatric SMA patients (Dobelmann et al., 2024). Our observation of increased THBS4 expression in DM1 suggests CNS involvement and potential to serve as a broader biomarker.

Src is a non-receptor protein tyrosine kinase widely expressed in the central nervous system that plays a crucial role in neuronal development and synaptic plasticity. It is essential for regulating pain transmission and is involved in signaling pathways of various neurological diseases, including migraine and neuropathic pain (Nie et al., 2021). We observed elevated Src levels in DM1, and studies indicate that Src inhibition is a promising target for treating chronic pain (Ge et al., 2020). Additionally, inhibiting Src has been shown to reduce neuroinflammation and protect dopaminergic neurons in Parkinson’s disease models (Yang et al., 2020). Interestingly, increased Src phosphorylation has been reported in models of spinal and bulbar muscular atrophy (SBMA), where Src inhibition improved disease phenotypes (Iida et al., 2019). While exploratory, our findings suggest that the identified proteins could serve as promising biomarkers for future studies. For SRC, our results show upregulation, which, consistent with SBMA studies, may reflect modulation of protein function relevant to disease pathways and phenotypes.

Furthermore, we observed dysregulation of angiogenic and inflammatory pathways in DM1, notably a decrease in ANGPT1 expression. ANGPT1 plays a key role in regulating the vascular endothelial barrier and its dysregulation has been implicated in chronic inflammation in Duchenne muscular dystrophy (DMD) (Lin et al., 2023). Our findings suggest that ANGPT1 dysregulation may also contribute to the pathological inflammation observed in DM1. Inflammation in DM1 was further supported by the elevated levels of pro-inflammatory cytokines IL-6 and IL-1ra in CSF, consistent with previous reports in other neuromuscular disorders such as DMD and SMA (Stephenson et al., 2020; Nuzzo et al., 2023). Interestingly, the reduced expression of CD59, a complement regulator, also suggests that impaired complement regulation may exacerbate inflammation and tissue damage in DM1 (Dalakas et al., 2020). Together, these findings indicate that the altered inflammatory response in DM1 could contribute to neurodegeneration and worsen CNS dysfunction.

We identified significant dysregulation in critical protein signaling pathways, including insulin signaling and MAPK pathways, which may play key roles in DM1 pathophysiology. These findings are consistent with previous investigations where impairments in protein signaling are well-established contributors to neuromuscular (Vainzof and Zatz, 2003) and CNS (Chico et al., 2009) dysfunction, muscle degeneration, and progression of various brain disorders (Réthelyi et al., 2023). Insulin resistance, a hallmark feature of metabolic syndrome in DM1, has been documented in over 30 clinical studies spanning six decades (Nieuwenhuis et al., 2019). Approximately 17% of DM1 patients experience insulin resistance, which is associated with significant cardiovascular issues, particularly cardiac failure (Boengler et al., 2017). Furthermore, reduced glucose uptake in the brain has been linked to cognitive deficits, including impairments in visuospatial and verbal memory (Annane et al., 1998; Okkersen et al., 2017). In preclinical models, DMPK knockout mice exhibit impaired insulin signaling and metabolic abnormalities, including abnormal glucose tolerance and decreased glucose uptake, suggesting defective intracellular trafficking of insulin and IGF-1 receptors (Llagostera et al., 2007). The role of insulin signaling in DM1 is further supported by clinical trials using metformin, which has shown promising effects on exercise capacity and mobility in DM1 patients (Bassez et al., 2018) and is established as an effective treatment for hyperglycemia in DM1 (Kouki et al., 2005). Our current CSF proteomic analysis complements these findings, revealing altered IGF transport in DM1 and further underscoring disrupted insulin signaling role in CNS function.

In addition to insulin signaling, we observed dysregulation of mitogen-activated protein kinases (MAPKs), which are central regulators of neuronal development, synaptic plasticity, and cell survival (Kim and Choi, 2010). P38 MAPKs, in particular, mediate stress and inflammation responses as well as myogenesis, and have been explored as therapeutic targets in several neurodegenerative and neuromuscular disorders, including facioscapulohumeral muscular dystrophy (FSHD) (Ahmed et al., 2020; Brennan et al., 2021). Additionally, p38 kinases are found in all major brain cell types and are involved in cognitive (Asih et al., 2020) and synaptic functions (Falcicchia et al., 2020). Our findings align with these studies, as we observed significant alterations in MAPK signaling pathways in DM1 patients, suggesting their involvement in disease progression. Recent studies have shown that inhibiting both PI3K/Akt and ERK MAPK pathways in SMA models reduced levels of SMN protein and mRNA while affecting mTOR phosphorylation and autophagy markers, revealing potential therapeutic targets for SMA (Nuzzo et al., 2023). Our observations of altered MAPK and PI3K/Akt signaling in DM1 suggest dysregulation of pathways like GSK3β-CUGBP1 (Lutz et al., 2023), which may contribute to disease pathophysiology and provide potential avenues for future therapeutic strategies targeting both the molecular underpinnings and clinical progression of DM1.

While our study provides the first CSF proteomic characterization of DM1, several limitations must be acknowledged. The small cohort size (*n* = 16) reflects the rarity of DM1 and challenges in CSF collection, resulting in adequate statistical power only for large effect sizes and limited generalizability due to the predominantly female sample (10/11 DM1 participants). Given known sex-based differences in immune and neuroinflammatory responses, replication in larger, sex-balanced, and phenotypically diverse cohorts including congenital and childhood-onset DM1 is essential. Another key limitation is the lack of standardized CTG repeat length measurements for all participants, which precluded correlation analyses between molecular and genetic disease severity. Since repeat size strongly influences clinical phenotype, future studies should incorporate precise repeat quantification (e.g., triplet-primed PCR or long-read sequencing) to explore genotype–proteotype relationships and identify severity-specific biomarkers. Additionally, our reliance on a single proteomic platform (Olink) limits cross-platform validation; complementary technologies such as SOMAscan, Simoa, or ELISA are needed to confirm our findings and rule out platform-specific effects. Validation in additional matrices (e.g., plasma, muscle, or brain tissue) would also clarify compartment specificity and assess blood–brain barrier permeability. Encouragingly, several proteins identified here, including THBS4, NCAM1, and IL-6, have been independently validated in related neuromuscular disorders, supporting their biological relevance. Finally, while our Olink panels covered 1,072 proteins, they did not capture splice variants, an important consideration given the splicing defects underlying DM1. Despite these limitations, our exploratory findings establish a foundational dataset for future multi-center validation, functional correlation, and therapeutic evaluation studies aimed at advancing CSF-based biomarkers for DM1 diagnosis and monitoring.

## Data Availability

The datasets generated during and/or analyzed during the current study are available from the corresponding author on reasonable request.

## Footnotes

1
https://olink.com/products/olink-target-96
