# Supplementary material for: Cerebrospinal fluid proteomic profiling reveals potential biomarkers and altered pathways in myotonic dystrophy type 1
Source: Front Neurosci. 2025 Nov 26;19:1709678. doi: 10.3389/fnins.2025.1709678 (PMC12689921; doi:10.3389/fnins.2025.1709678)
Supplement: Supplementary file 1 [file Table_1.docx]

**Supplementary Table S1:** Post-hoc statistical power analysis for the six differentially expressed proteins identified in CSF from DM1 patients vs. healthy controls. Power calculations were performed using observed effect sizes (Cohen's d), current sample sizes (n=11 DM1, n=5 HC), and α=0.05 (two-tailed). NPX = Normalized Protein eXpression. Sample size calculations indicate the total number of participants needed to achieve 80% power at the observed effect size.

| **Protein** | **Log2 Fold Change** | **Mean DM1 (NPX)** | **Mean HC (NPX)** | **Cohen's d** | **Statistical Power (%)** | **Sample Size Needed for 80% Power** |
| --- | --- | --- | --- | --- | --- | --- |
| CD59 | 2.5 | 5.30 | 6.36 | 2.1 | 82 | 12 (6 per group) |
| CKAP4 | 2.2 | 2.76 | 3.74 | 1.9 | 75 | 14 (7 per group) |
| NCAM1 | 1.8 | 7.27 | 8.22 | 1.6 | 65 | 18 (9 per group) |
| SCARF1 | 1.5 | 4.46 | 5.15 | 1.3 | 52 | 26 (13 per group) |
| PTH1R | 1.3 | 1.23 | 1.86 | 1.1 | 45 | 34 (17 per group) |
| CA4 | 1.2 | 5.61 | 6.43 | 1.0 | 38 | 42 (21 per group) |
